# iTRAQ-Based Identification of Proteins Related to Muscle Growth in the Pacific Abalone, *Haliotis discus hannai*

**DOI:** 10.3390/ijms18112237

**Published:** 2017-10-25

**Authors:** Jianfang Huang, Weiwei You, Xuan Luo, Caihuan Ke

**Affiliations:** 1 State Key Laboratory of Marine Environmental Science, Xiamen University, Xiamen 361102, China; jianfhuang@foxmail.com (J.H.); xluo@xmu.edu.cn (X.L.); 2 College of Ocean and Earth Sciences, Xiamen University, Xiamen 361102, China; 3 Fujian Collaborative Innovation Center for Exploitation and Utilization of Marine Biological Resources, Xiamen University, Xiamen 361102, China

**Keywords:** *Haliotis discus hannai*, iTRAQ, growth-related protein, molecular mechanisms, abalone

## Abstract

The abalone *Haliotis discus hannai* is an important aquaculture species that is grown for human consumption. However, little is known of the genetic mechanisms governing muscle growth in this species, particularly with respect to proteomics. The isobaric tag for relative and absolute quantitation (iTRAQ) method allows for sensitive and accurate protein quantification. Our study was the first to use iTRAQ-based quantitative proteomics to investigate muscle growth regulation in *H. discus hannai*. Among the 1904 proteins identified from six samples, 125 proteins were differentially expressed in large specimens of *H. discus hannai* as compared to small specimens. In the large specimens, 47 proteins were upregulated and 78 were downregulated. Many of the significant Kyoto Encyclopedia of Genes and Genomes (KEGG) pathways, including these differentially expressed proteins, were closely related to muscle growth, including apoptosis, thyroid hormone signaling, regulation of the actin cytoskeleton, and viral myocarditis (*p* < 0.05). Our quantitative real-time polymerase chain reaction (qRT-PCR) analyses suggested that the alterations in expression levels observed in the differentially expressed proteins were consistent with the alterations observed in the encoding mRNAs, indicating the repeatability of our proteomic approach. Our findings contribute to the knowledge of the molecular mechanisms of muscle growth in *H. discus hannai*.

## 1. Introduction

The Pacific abalone, *Haliotis discus hannai*, is the most popular cultivated abalone throughout China, Japan, and South Korea; it is known for its delicate flavor and high market value [1]. To meet increasing commercial demand, large-scale aquaculture of the Pacific abalone in China began in the late 1980s [1,2]. Moreover, abalone farming has gradually extended from the northern Yellow Sea to the East China Sea [3], and now Fujian province accounts for nearly 80% (112,611 tons/139,697 tons) of total abalone production in China [4]. The time required to grow the abalone to market size is the most important factor contributing to abalone production efficiency [5]. Because the edible portion of the abalone is the foot muscle, genetic improvement programs in the abalone industry have typically focused on enhancing muscle growth as well as increasing growth speed [6]. Unfortunately, little is yet known about the genes involved in muscle growth in *H. discus hannai*. A better understanding of the genetic mechanisms involved in muscle growth in *H. discus hannai* is critical for efficient selective breeding.

Proteomic profiling, a direct reflection of gene expression patterns, provides information about gene regulation and active pathways. Most previous studies of gene expression in marine invertebrates have relied on two-dimensional gel electrophoresis, a technique that is limited by low throughput and low reproducibility, such as abalone [7], brine shrimp [8], marine snail [9], and *Pomacea canaliculata* [10]. In contrast, recently developed proteomic techniques, such as isobaric tags for relative and absolute quantitation (iTRAQ), provide more reliable quantitative measurements and allow for comparisons among samples [11]. Indeed, iTRAQ, used in combination with two-dimensional liquid chromatography and tandem mass spectrometry (LC-MS/MS) is one of the most reliable methods of quantitative proteomic analysis available to date [12,13,14], especially in aquatic animals, such as medaka fish [15], *Urobatis jamaicensis* [16], zebrafish [17], *Crassostrea gigas* [18], *Portunus trituberculatus* [19], and *Apostichopus japonicus* [20].

Here, we used iTRAQ with GO (Gene Ontology) functional annotations, and KEGG (Kyoto Encyclopedia of Genes and Genomes) pathway information to assess the proteomic changes in the muscle tissues of 2-year-old specimens of *H. discus hannai*. Our aim was to identify the specific proteins involved in muscle growth regulation in both fast- and slow-growing individuals. We identified several growth-related proteins involved in diverse biological processes, including ion binding, protein binding, metabolism, and stress response. We performed qRT-PCR validation experiments to detect the expression levels of the genes encoding some representative proteins in various tissues. Our results increase the knowledge of the mechanisms underlying abalone muscle growth.

## 2. Results

### 2.1. Protein Profiling

We generated 425,477 spectra from the six samples analyzed. Of these, 1904 proteins (Table S1) were quantified with Mascot, based on 44,436 high-confidence spectra (Table 1).

### 2.2. Differentially Expressed Proteins (DEPs)

**Figure 1:**
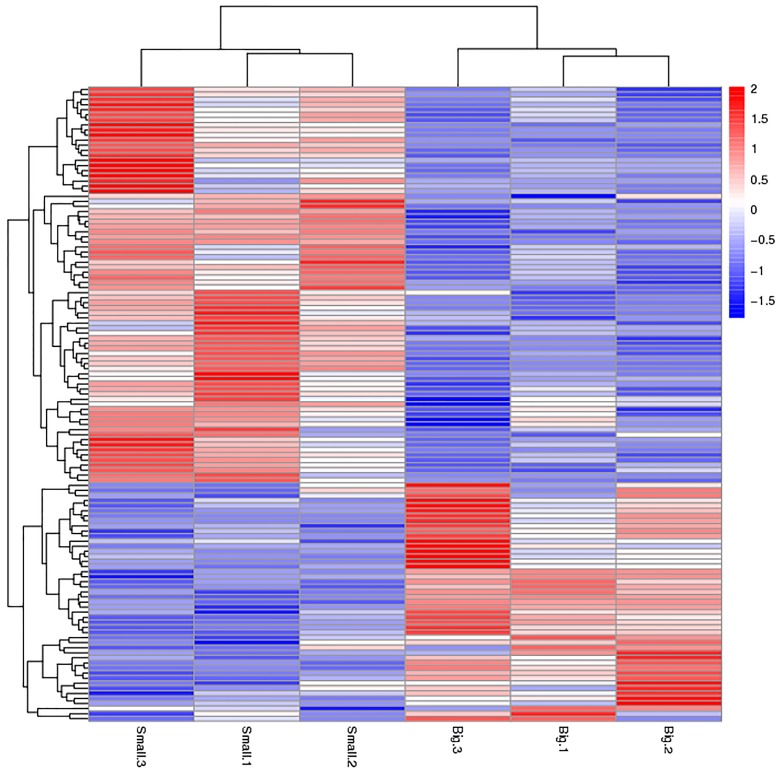
Hierarchical clustering analysis of the two size classes of abalone analyzed, showing the proteins differentially expressed between small and large specimens of *Haliotis discus hannai*. Red and blue colored bars indicate up- and down-regulation, respectively.

**Figure 2:**
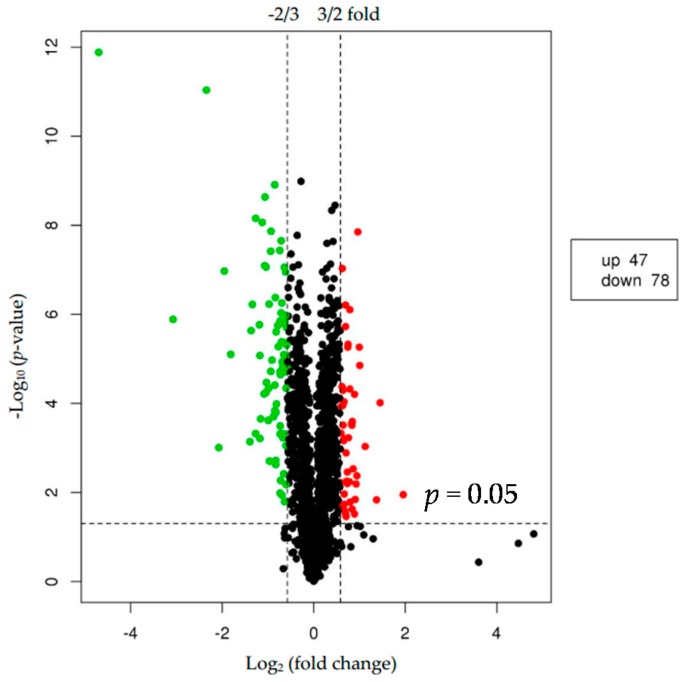
Volcano plot showing proteins differentially expressed between the two groups of abalone (larger and smaller). The upregulated and downregulated proteins (*p* < 0.05 for both) are shown in red and green, respectively. Black represents no significant change in expression level.

We used *p* < 0.05 as a threshold indicating a significant alteration in protein expression. We further quantified 125 significant DEPs with iTRAQ (Tables S2 and S3). In the larger abalones, 47 proteins were upregulated, and 78 were downregulated compared to the smaller abalones. Hierarchical clustering analysis based on differences in protein expression recovered a clear distinction between the larger abalones and the smaller ones (Figure 1 and Figure 2).

### 2.3. GO Functional Classification

Based on our analysis of the GO database, some of our identified proteins are involved in various biological functions: metabolic process (98 proteins), intracellular protein transport (73 proteins), or proteolysis (45 proteins; Figure S1). Others are categorized as cellular components: membrane (73 proteins) or microtubule (70 proteins; Figure S1). The remaining proteins are involved in major molecular functions: protein binding (3240 proteins) and calcium ion binding (614 proteins; Figure S1).

**Figure 3:**
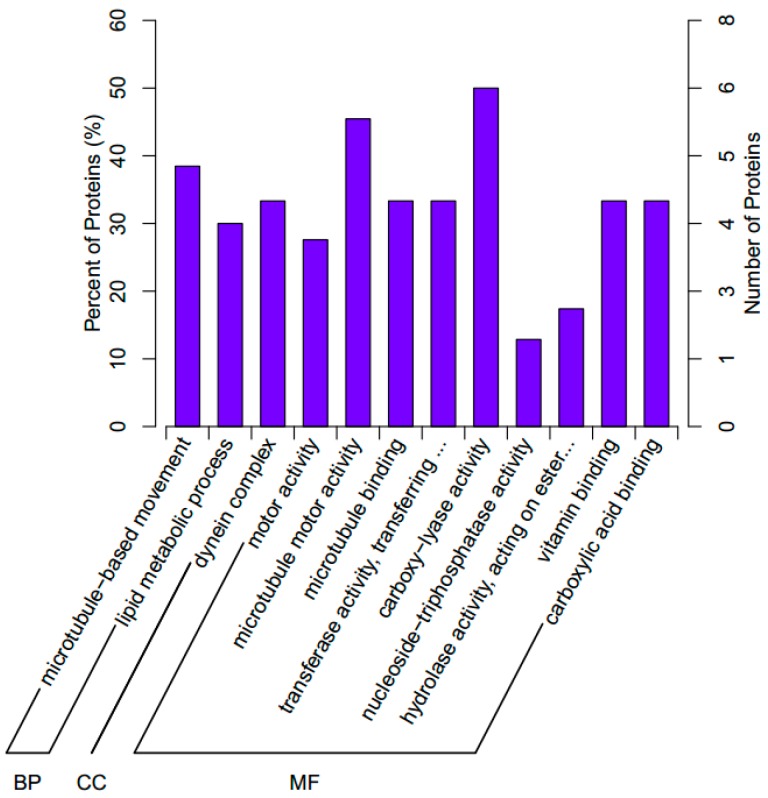
The Gene Ontology (GO) classifications assigned to the significant differentially expressed proteins in the abalone *H. discus hannai*. BP, biological processes; CC, cellular components; MF, molecular functions.

We further investigated the biological processes associated with the significant DEPs. In short, 12 GO terms were assigned to the significant DEPs in three independent ontological categories: biological processes (2 terms), cellular components (1 term), and molecular function (9 terms; Figure 3).

### 2.4. KEGG Pathway Analysis

Our KEGG pathway enrichment analysis recovered 11 significant pathways (*p* < 0.05; Table 2), including lysosome (eight proteins), adherens junction (seven proteins), bladder cancer (three proteins), and apoptosis (seven proteins). Two additional significant pathways may be involved in growth regulation in *H. discus hannai*: the thyroid hormone signaling pathway (six proteins), and the regulation of the actin cytoskeleton (nine proteins).

In the apoptosis KEGG pathway, four proteins (actin A1, myophilin, actin-2, and actin) were upregulated in the larger abalones, and three proteins (CTSC, GDPD1, CTSB) were downregulated. In the thyroid hormone signaling pathway, the majority of the enriched proteins (actin A1, SLC2A3, actin-2, and actin) were upregulated in the larger abalones, although the enriched proteins ACTB and GDPD1 were downregulated. In contrast, in the actin cytoskeleton regulation pathway, the upregulated proteins in the larger abalones were actin A1, actin-2, and actin, while profilin, ACTB, PIP4K2B, EGFR, GDPD1, and the myosin regulatory light chain sqh were downregulated. Across all significant KEGG pathways, more proteins were downregulated in the large specimens of *H. discus hannai* than in the small specimens.

### 2.5. qRT-PCR

**Figure 4:**
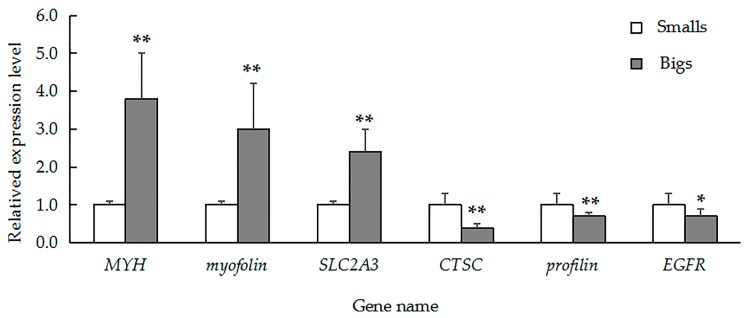
Quantitative real-time polymerase chain reaction (QRT-PCR) analysis comparing relative gene expression among *MYH*, *myophilin*, *SLC2A3*, *CTSC*, *profilin*, and *EGFR* in large and small specimens of *H. discus hannai.* Gene expression was normalized to *18S rRNA*. The data are expressed as means ± SD (*n* = 3). Large specimens, grey bars; small specimens, white bars. Asterisks represent statistically significant differences. * and ** indicate *p* < 0.05 and *p* < 0.01, respectively.

**Figure 5:**
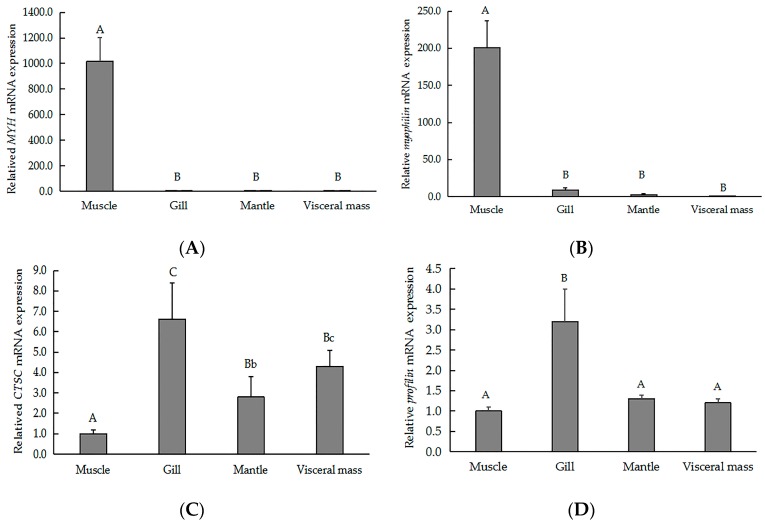
QRT-PCR analysis indicating the relative gene expression of (**A**) *MYH*, (**B**) *myophilin*, (**C**) *CTSC*, and (**D**) *profilin* across various tissues of the of *H. discus hannai*. Gene expression levels were normalized to *18S rRNA*. Data are expressed as the means ± SD of at three biological replicates. Different letters represent statistically significant differences. Lowercase and capital letters indicate *p* < 0.05 and *p* < 0.01, respectively.

To validate our iTRAQ results and to analyze the correlation between gene and protein expression, we used qRT-PCR to quantify the expression of the genes that encode six previously identified representative DEPs. We selected three genes that encoded upregulated proteins (myophilin, SLC2A3, and MYH) and three genes that encoded downregulated proteins (CTSC, EGFR, and profilin). We found that the level of mRNA expression varied in parallel to the corresponding protein expression (Figure 4). We also found that four gene transcripts were nearly expressed across all of the tissues (muscle, gill, visceral mass, and mantle). The highest levels of *MYH* (Figure 5A) and *myophilin* (Figure 5B) gene expression were found the muscle; expression levels of *MYH* and the *myophilin* gene were extremely low in the gill, visceral mass, and mantle (Figure 5A,B). However, the expression levels of the genes *CTSC* (Figure 5C) and *profilin* (Figure 5D) were lowest in the muscle tissue.

## 3. Discussion

Body weight is a complex trait that is regulated by the coordinated activity of several proteins. According to the previous study [21], we believe that larger abalones have higher growth rate; on the contrary, smaller individuals with low growth rate. So this provides material for the study of abalone growth. The abalones are divided into small-, medium-, and large-size groups to identify genes associated with high growth rates using RNA sequencing technology [22]. To further clarify the mechanisms underlying growth regulation, we used an iTRAQ-based quantitative proteomic method to compare large and small specimens of the abalone *H. discus hannai*. To our knowledge, this is the first study to analyze growth mechanisms in the abalone using this method. We identified 125 proteins that were expressed differently in the larger abalones as compared to the smaller abalones: 47 were upregulated and 78 were downregulated. These DEPs were shown to be involved in multiple biological processes and signaling pathways, including apoptosis, thyroid hormone signaling, and the regulation of the actin cytoskeleton. These processes are related to muscle growth.

In molluscs, muscle protein synthesis is very important for growth. Here, we identified several proteins involved in molluscan muscle growth, including the myosin II heavy chain (MYH II) and MYH; previous research has indicated that the *myosin* gene is positively correlated with muscle development [23,24]. The *myosin heavy chain 67* was expressed in many bovine tissues, including the adductor muscle, the longissimus dorsi, the psoas, the atrium, the ventricle, the aorta, and the liver [25]. In the *Siniperca chuatsi*, a freshwater fish species, the highest level of MYH-7b protein expression is found in red muscle, followed by the skin, pituitary, and eyes, while the lowest levels are found in the myocardium, gills, and gonads [26]. In the Atlantic salmon, the highest MYH expression always contain higher levels of muscle protein [23]. Here, we found the highest level of *MYH* gene expression in the muscle, suggesting that the MYH protein may play an important role in muscle development in *H. discus hannai*. The protein expression levels of MYH and MYH II were upregulated in the larger abalones when compared to the smaller abalones, suggesting that differences in size might be correlated with properties of the foot tissue, including glandular or accessory foot organs [27,28]. Together, our results suggest that the proteins MYH and MYH II are important regulatory factors in molluscan muscle growth, and should be the focus of further studies of growth in *H. discus hannai*.

All actins are globular, microfilament-forming, multi-functional proteins; they are highly conserved among almost all eukaryotes [29]. In vertebrates, muscular and non-muscular actins are distinguished by characteristic differences in amino acid composition [30]. In invertebrates, actins are also classed as muscular or non-muscular, but the two classes are not distinguishable based on amino acid sequence [30]. Members of the *actin* gene family are involved in various cellular processes, including muscle contraction, cell motility, cell division and cytokinesis, vesicle and organelle movement, cell signaling, and the establishment and maintenance of cell junctions, and cell shape [31]. To date, *actin* genes have been isolated from several molluscs, including *Aplysia californica* [32,33], *Crassostrea gigas* [34], *Placopecten magellanicus* [30], and *Patella vulgata* [35]. It is suggest that the *actin* gene family of the abalone *H. iris* include at least eight genes; three of these, *H. iris A*1, *H. iris A*2, and *H. iris A*3, are shown to be differentially expressed [36]. Here, we found that four actin proteins (actin A1, actin-2, actin, and ACTB) were differentially expressed in the larger abalones as compared to the smaller abalones. Our KEGG pathway analysis indicated that these DEPs were primarily involved in the adherens junction, in the thyroid hormone signaling pathway, in viral myocarditis, and in the regulation of the actin cytoskeleton. These results were consistent with previous work. However, the functions of the actin proteins that we identified in *H. discus hannai* were unknown, which is indicative of the complexity of the functions of actin proteins in abalone muscle growth.

Profilin, an actin-binding protein, is involved in the dynamic turnover and restructuring of the actin cytoskeleton [37]. In some cases, profilin binds to monomeric actin, thereby inhibiting polymerization, while under other conditions it stimulates polymerization. Profilin is present in almost all mature mouse tissue, with the exception of skeletal muscle [38]. The binding of profilin to barbed ends inhibits filament branching by both WASP proteins and the Arp2/3 complex, resulting in actin-based motility [39]. The injection of profilin inhibits both lamellipodium motility and the formation of the lamellipodial branched filaments [40,41]. Profilin also reinforces the complex relationships among RTK pathways, membrane lipids, actin-binding proteins, and the actin cytoskeleton [42]. Consistent with previous studies, we found that the protein expression of profilin was downregulated in the larger abalones as compared to the smaller abalones; overall, the lowest profilin expression was found in the muscle [38]. Therefore, we speculate that molluscan profilin may be involved in the regulation of the actin cytoskeleton through dynamic control of actin polymerization, consequently influencing muscle growth.

Metabolic alterations are tightly associated with physiological adaptations [16]. Here, many of the DEPs (Tables S2 and S3) were enzymes implicated in lipid metabolism or other catalytic activities, including both upregulated proteins (e.g., chitin synthase, dynein heavy chain 10, propionyl-CoA carboxylase β chain, and 2-amino-3-carboxymuconate-6-semialdehyde decarboxylase) and downregulated proteins (e.g., *N*-acetylglucosamine-6-sulfatase, carbohydrate sulfotransferase 11, and thymidine phosphorylase). Some proteins implicated in DNA duplication and protein synthesis (e.g., leucine-rich repeat-containing protein 9, ganglioside-induced differentiation-associated protein 1, and 40S ribosomal protein S12) were upregulated, while others (e.g., glutathione-*S*-transferase Mu 2, transgelin, lymphocyte cytosolic protein 2, and rho GTPase-activating protein 44) were downregulated. Therefore, our data indicate that, to promote growth in larger specimens of abalone, more energy is conserved than in smaller specimens, and available resources are distributed differently.

A number of apoptosis-related proteins (e.g., myophilin, CTSC, and CTSB). Myophilin is first identified as a muscle-specific protein in *E. granulosus* and is involved in the regulation of muscle contraction [43,44]. A myophilin-like protein highly similar to myophilin was subsequently identified in *E. granulosus*. The rsj-myophilin-like protein may be useful as an adjunct immunodiagnostic antigen for schistosomiasis, as well as a marker of drug treatment efficacy [45]. CTSB is a lysosomal cysteine protease in the papain superfamily; it plays an important role in intracellular proteolysis [46]. Upregulation of CTSB was found in various cancers as well as in premalignant lesions and other pathological conditions [47,48,49]. CTSB is produced by the muscle tissue during metabolism [50]. *CTSB* gene knockout experiments showed that CTSB was involved in epilepsy-related apoptotic cell death [51]. CTSC is a lysosomal exo-cysteine protease and a member of the papain superfamily [52]. The enzyme is first synthesized as procathepsin C, is modified into mature CTSC, and then appears to oligomerize just before entry into the lysosome [53,54,55]. CTSC appears to coordinate the activation of many serine proteases in immune and inflammatory cells. CTSC also activates the granzymes, which are the main effector proteases that are involved in NK cell cytotoxicity [56]. Here, both CTSB and CTSC were downregulated in the larger specimens, while the myophilin protein was upregulated. Both *CTSC* and *myophilin* were expressed across all of the examined tissues (muscle, gill, visceral mass, and mantle); these proteins were both involved in the lysosome and the apoptosis pathways. We speculate that the reduced expression of CTSB and CTSC and the overexpression of the myophilin protein not only reflect the decreased levels of cell apoptosis found in the larger specimens, but also indicate that these proteins play important roles in the growth of *H. discus hannai*.

Here, we detected several proteins that were uncharacterized, as well as proteins with unknown functions in muscle growth. Our results indicate the complexity of growth mechanisms in gastropod molluscs. The DEPs identified in this study may serve as candidate biomarkers for further studies of molluscan muscle growth. However, further studies are needed to confirm our results, and the mechanisms involving muscle growth-related proteins require further clarification.

## 4. Materials and Methods

### 4.1. Experimental Tissue

Since 2014, a breeding population of *H. discus hannai* has produced pedigreed offspring; all of the matings are controlled, and are conducted at Fuda Aquiculture in Jinjiang, Fujian province, China. The six specimens used in this study were obtained from this facility at 2 years of age. Three of the specimens were smaller (“Smalls” group; mean weight, 16.5 ± 1.0 g), and three were larger (“Bigs” group; mean weight, 95.1 ± 7.7 g). All six acclimated in our laboratory for 1 week before dissection. Samples of the muscle, gill, visceral mass, and mantle were collected from each specimen, immediately snap-frozen in liquid nitrogen, and stored at −80 °C.

### 4.2. Protein Preparation

The muscle tissue samples were individually milled to a power in a mortar with liquid nitrogen. We then mixed 150 mg of the powder from each sample with 1 mL of lysis buffer containing Tris-base (pH 8), 7 M Urea, 2 M Thiourea, 0.1% SDS, 2 mM EDTA, complete protease inhibitor cocktail (Sigma, St. Louis, MO, USA), and 1 mM phenylmethylsulfonyl fluoride in a glass homogenizer. The homogenate was incubated on ice for 20 min and then centrifuged at 12,000× *g* for 15 min at 4 °C. The supernatant was transferred to a clean tube, and protein concentration was determined with a Bradford assay.

### 4.3. iTRAQ Labeling of Peptides

The supernatant from each sample, containing precisely 100 μg of protein, was digested with Trypsin Gold (Promega, Madison, WI, USA) at 37 °C for 16 h. After trypsin digestion, peptides were dried by vacuum centrifugation. Desalted peptides were labeled with iTRAQ reagents (iTRAQ^®^ Reagent-8PLEX Multiplex Kit, Sigma), following the manufacturer’s instructions (AB Sciex, Foster City, CA, USA). For 100 μg of peptide, 1 unit of labeling reagent was used. Peptides were dissolved in 20 µL of 0.5 M triethylammonium bicarbonate solution (TEAB, pH 8.5), and the labeling reagent was added to 70 µL of isopropanol. After incubation for 1 h, the reaction was stopped with 50 mM Tris/HCl (pH 7.5). The labeled peptides were incubated at 25 °C for 2 h. Differently labeled peptides were mixed and then desalted in 100 mg SCX columns (strata-x-c, Phenomenex: 8B-S029-EBJ).

### 4.4. HPLC Fractionation

The first dimension RP separation by microLC was performed on an L-3000 HPLC System (Rigol, Beijing, China) by using a Durashell RP column (5 μm, 150 Å, 250 mm × 4.6 mm i.d., Agela, Wilmington, DE, USA). Mobile phases A (2% acetonitrile plus 20 mM NH_4_FA; pH adjusted to 10.0 with NH_3_·H_2_O) and B (98% acetonitrile plus 20 mM NH_4_FA; pH adjusted to 10.0 with NH_3_·H_2_O) were used to develop a gradient elution. The solvent gradient was as follows: 5–8% B, 2 min; 8–18% B, 11 min; 18–32% B, 9 min; 32–95% B, 1 min; 95% B, 1 min; 95–5% B, 2 min. Tryptic peptides were separated at an eluent flow rate of 1 mL/min and monitored at UV 214 nm. The column oven was set at 37 °C. Eluent was collected every minute and was merged to generate 10 fractions. Samples were vacuum dried and then reconstituted in 15 μL water containing 0.1% (*v*/*v*) FA and 5% (*v*/*v*) acetonitrile.

### 4.5. LC-MS/MS Analysis

Fractions from the first dimension RPLC were dissolved with loading buffer and then separated with a C18 column (75 μm inner-diameter, 360 μm outer-diameter × 10 cm, 3 μm C18). Mobile phase A was a solution of 0.1% formic acid in water, and mobile phase B was a solution of 0.1% formic acid in acetonitrile; we applied a series of adjusted linear gradients with a flow rate of 300 nL/min, based on the hydrophobicity of fractions eluted in 1D LC.

We then used an Orbitrap Q-Exactive for MS analysis. The source operated at 1.8 kV. For the full MS survey scan, the AGC target was 3e6, and the scan range was 350 to 1800 with a resolution of 70,000. For iTRAQ-labeled peptide, the top 20 peaks with charge state 2 and above were selected for fragmentation by HCD with normalized collision energy of 30%. The resolutions of the MS2 spectra were set to 17,500.

### 4.6. Data Analysis

We first searched the raw MS data files generated by Q-Exactive with Mascot 2.6.0 (Matrix Science, London, UK). Mascot was used to search the protein database (*H. discus hannai*, 29,385 entries) that derived from the annotation of de novo genome assembly, assuming the digestion enzyme trypsin. Carbamidomethyl of cysteine and iTRAQ8plex of lysine and the n-terminus were specified in Mascot as fixed modifications. Oxidation of methionine, acetyl of the n-terminus and iTRAQ8plex of tyrosine were specified in Mascot as variable modifications. Protein identifications were accepted if they contained at least one identified peptide and the probability of an FDR was less than 10.0% in Scaffold software (version Scaffold_4.0.7, Proteome Software Inc., Portland, OR, USA). We only used significant ratios, defined as *p* < 0.05 and |log_2_FC| > 0.585. We used the databases GO (http://www.geneontology.org) and KEGG (http://www.genome.jp/kegg/) to predict gene function.

### 4.7. qRT-PCR

Six genes were selected for confirmation and analysis with qRT-PCR. Gene-specific primers (Table S4) were designed with Primer Premier 3.0. Total RNA was extracted from the previously collected tissues (muscle, gill, visceral mass, and mantle) of all the specimens. Total RNA was then reverse-transcribed into cDNA with a PrimeScript RT Reagent Kit (TaKaRa, Dalian, China). The qRT-PCR reaction was performed in a 20 μL volume using a Faststart Universal SYBR Green Master (Rox) (Roche), 120 nM of each specific primer, and 5 μL of cDNA. We used the following cycling protocol: 95 °C for 10 min; 40 cycles of 95 °C for 15 s and 59 °C for 30 s; followed by a melting curve. All of the qRT-PCRs were run in triplicate. The 2^−∆∆*C*t^ method was used to analyze the differences in gene expression [57].

### 4.8. Statistical Analysis

Statistical analyses were performed with SPSS 19.0. Data shown are the mean ± SD of three replicates. Statistical significance was evaluated using the Student’s *t* test. A *p*-value < 0.05 was considered statistically significant.

## 5. Conclusions

Here we present the first report of differentially expressed proteins in large versus small specimens of *H. discus hannai* using the iTRAQ technique. Interestingly, we discovered that many of DEPs we identified were involved in apoptosis, thyroid hormone signaling, the regulation of actin cytoskeleton, and other signaling pathways. Some of these DEPs and pathways were closely related to muscle growth. However, further investigation is required to confirm the predicted roles of these identified proteins. Our results deepen our understanding of the mechanisms underlying muscle growth in the abalone.

## Figures and Tables

**Table 1:** Overview of the proteomics sequencing results.

Group Name	Total Spectra	Spectra	Ratio Identified	Peptides	Proteins
All	425,477	44,436	10.40%	10,097	1904

**Table 2:** Proteins differentially expressed in the large specimens of *H. discus hannai*, as compared to the small specimens in various significant Kyoto Encyclopedia of Genes and Genomes (KEGG) pathways.

KEGG Pathway	Upregulated Proteins	Downregulated Proteins
Lysosome	putative inorganic phosphate cotransporter (Picot; Accession Number: O61369), actin (Accession Number: Q93129)	ganglioside GM2 activator (GM2A; Accession Number: Q8HXX6), cathepsin C (CTSC; Accession Number: A0A023PJH7), cathepsin B (CTSB; Accession Number: A1E295), palmitoyl-protein thioesterase 1 (PPT1; Accession Number: Q8HXW6), *N*-acetylglucosamine-6-sulfatase (GNS; Accession Number: Q8BFR4), α-*N*-acetylgalactosaminidase (Accession Number: Q90744)
Adherens junction	actin A1 (Accession Number: Q5BQE5), actin-2 (Accession Number: P26197), actin	kitasatospora griseola strain MF730-N6 RKJC_4 (Accession Number: A0A0D0PVQ3), β actin (ACTB; Accession Number: G8HY07), epidermal growth factor receptor (EGFR; Accession Number: P0CY46), glycerophosphodiester phosphodiesterase domain-containing protein 1 (GDPD1; Accession Number: Q8N9F7)
Bladder cancer	-	thymidine phosphorylase (Tymp; Accession Number: Q5FVR2), EGFR, GDPD1
Apoptosis	actin A1, actin-2, actin, myophilin (Accession Number: Q24799)	CTSC, GDPD1, CTSB
Thyroid hormone signaling pathway	actin A1, actin-2, actin, solute carrier family 2 facilitated glucose transporter member 3 (SLC2A3; Accession Number: P47843)	ACTB, GDPD1
Endometrial cancer	-	kitasatospora griseola strain MF730-N6 RKJC_4, EGFR, GDPD1
Shigellosis	actin A1, actin-2, actin	profilin (Accession Number: F4XXT7), ACTB, GDPD1
Regulation of actin cytoskeleton	actin A1, actin-2, actin	profilin, ACTB, EGFR, GDPD1, phosphatidylinositol 5-phosphate 4-kinase type-2 β (PIP4K2B; Accession Number: P78356), myosin regulatory light chain sqh (Accession Number: P40423)
Salmonella infection	actin A1, actin-2, actin	profilin, ACTB, GDPD1
Viral myocarditis	actin A1, actin-2, actin, myosin heavy chain (MYH; Accession Number: P24733), MYH II (Accession Number: O96700)	ACTB
Hippo signaling pathway-fly	actin A1, actin-2, actin, protocadherin Fat 4 (FAT4; Accession Number: Q6V0I7)	ACTB

## Supplementary Materials

Supplementary materials can be found at www.mdpi.com/1422-0067/18/11/2237/s1.
