# Supplementary material for: iTRAQ-Based Identification of Proteins Related to Muscle Growth in the Pacific Abalone, Haliotis discus hannai
Source: Int J Mol Sci. 2017 Oct 25;18(11):2237. doi: 10.3390/ijms18112237 (PMC5713207; doi:10.3390/ijms18112237)
Supplement: Supplementary file 1 [file ijms-18-02237-s001.zip › ijms-230384 final supplementary-new/ijms-230384 final supplementary.docx.docx]

Supplementary Materials: iTRAQ-Based Identification of Proteins Related to Muscle Growth in the Pacific Abalone, *Haliotis discus hannai*

**Jianfang Huang, Weiwei You, Xuan Luo, Caihuan Ke**

**Table S2.** Upregulated proteins in the muscle of the *Haliotis discus hannai*

| **Protein Description** | **Accession Number** | **Coverage (%)** | **Peptide** | ***p*-value** |
| --- | --- | --- | --- | --- |
| **ion binding** | | | | |
| myosin II heavy chain | O96700 | 28.8 | 5 | 5.44E-06 |
| Adenylate kinase isoenzyme 1 | Q20140 | 42.2 | 1 | 0.006440933 |
| Myosin heavy chain, striated muscle | P24733 | 18 | 2 | 0.006041034 |
| Kinesin-like protein KIF27 | Q7M6Z5 | 0.6 | 1 | 0.000252953 |
| Erythronolide synthase, modules 3 and 4 | Q03132 | 0.39 | 1 | 0.00420328 |
| Genome sequencing data, contig C301 | A8YEV4 | 50.6 | 8 | 0.000309076 |
| Procollagen-lysine,2-oxoglutarate 5-dioxygenase 1 | P24802 | 9.48 | 6 | 9.34E-08 |
| Tyrosine decarboxylase | Q95ZS2 | 2.97 | 2 | 0.006307755 |
| CCHC-type zinc finger protein CG3800 | Q8T8R1 | 6.67 | 1 | 0.002938947 |
| Protocadherin Fat 4 | Q6V0I7 | 0.77 | 1 | 0.030458943 |
| Chitin synthase | A5HKN1 | 0.3 | 1 | 0.000468375 |
| **protein binding** | | | | |
| Ankyrin repeat and SAM domain-containing protein 6 | P0C0T2 | 9.46 | 1 | 0.000114623 |
| Leucine-rich repeat-containing protein 9 | Q8CDN9 | 0.63 | 1 | 4.76E-05 |
| Myophilin | Q24799 | 54.7 | 8 | 6.19E-07 |
| Synaptotagmin-C | P24507 | 1.43 | 1 | 0.034573648 |
| Ganglioside-induced differentiation-associated protein 1 | Q8TB36 | 26.6 | 8 | 4.70E-06 |
| Cilia- and flagella-associated protein 70 | Q5T0N1 | 0.66 | 1 | 1.41E-08 |
| **hydrolase activity** | | | | |
| Dynein heavy chain 10, axonemal | Q8IVF4 | 0.48 | 1 | 6.25E-05 |
| **transport** | | | | |
| Solute carrier family 2, facilitated glucose transporter member 3 | P47843 | 5.47 | 4 | 0.001292446 |
| Proton-coupled folate transporter | Q7ZWG6 | 1.45 | 1 | 0.014638442 |
| Putative inorganic phosphate cotransporter | O61369 | 3.21 | 1 | 0.011184834 |
| Multidrug resistance-associated protein 1 | Q864R9 | 0.66 | 1 | 0.000591912 |
| Glutamine--fructose-6-phosphate aminotransferase [isomerizing] 1 | P47856 | 3.7 | 2 | 5.09E-05 |

**Table S2.** *cont.*

| **Protein Description** | **Accession Number** | **Coverage (%)** | **Peptide** | ***p*-value** |
| --- | --- | --- | --- | --- |
| **transferase activity** | | | | |
| Beta-1,3-galactosyltransferase 4 | Q9Z0F0 | 6.75 | 1 | 0.020151502 |
| **primary metabolic process** | | | | |
| Elongation factor 1-beta | P30151 | 17.3 | 3 | 0.000111876 |
| **intracellular** | | | | |
| Histone H4 | P0CG89 | 39.8 | 4 | 1.11E-07 |
| Dynein heavy chain 3, axonemal | Q8TD57 | 0.21 | 1 | 0.000283147 |
| 40S ribosomal protein S12 | P84175 | 14.4 | 2 | 9.22E-05 |
| **response to stress** | | | | |
| Uncharacterized protein C167.05 | P87132 | 16.5 | 2 | 7.87E-07 |
| Universal stress protein YxiE | P42297 | 9.7 | 1 | 0.023591567 |
| **ligase activity** | | | | |
| Propionyl-CoA carboxylase beta chain, mitochondrial | P79384 | 1.48 | 1 | 5.46E-06 |
| **lyase activity** | | | | |
| 2-amino-3-carboxymuconate-6-semialdehyde decarboxylase | Q8TDX5 | 3.99 | 1 | 0.000925744 |
| **Unknown function protein** | | | | |
| Phosducin-like protein 3 | Q5RB77 | 4.62 | 1 | 0.005746209 |
| Coiled-coil domain-containing protein 171 | E9Q1U1 | 0.58 | 1 | 0.005587162 |
| Uncharacterized protein | K1PMR8 | 19.3 | 2 | 0.005587162 |
| Uncharacterized protein | W4YJI7 | 0.49 | 1 | 0.010821754 |
| Uncharacterized protein | V4AFC5 | 0.99 | 1 | 0.00030745 |
| Actin | Q93129 | 33.7 | 1 | 0.000686045 |
| Actin A1 | Q5BQE5 | 46.5 | 30 | 1.41E-05 |
| 3-hydroxyacyl-CoA dehydrogenase type-2 | O18404 | 4.38 | 1 | 0.000627256 |
| Crystallin J1C | P40821 | 4.35 | 1 | 0.018523851 |
| Macrophage migration inhibitory factor | Q1ZZU7 | 12.5 | 1 | 0.016387702 |
| Neoverrucotoxin subunit alpha-like | V2WX82 | 5.46 | 1 | 0.030336043 |
| Actin-2 | P26197 | 7.41 | 1 | 0.014324748 |
| Uncharacterized protein | A8A947 | 0.94 | 2 | 0.026123339 |
| DnaJ homolog subfamily C member 1 | Q61712 | 1.4 | 1 | 0.003481557 |
| Uncharacterized protein | A0A0B7BLC7 | 84.5 | 10 | 1.88E-06 |

**Table S3.** Downregulated proteins in the muscle of the *Haliotis discus hannai*

| **Protein Description** | **Accession Number** | **Coverage (%)** | **Peptide** | ***p*-value** |
| --- | --- | --- | --- | --- |
| **ion binding** | | | | |
| Vitamin D(3) 25-hydroxylase | O46658 | 7.58 | 1 | 0.001906949 |
| Glypican-6 | Q5RE54 | 21 | 1 | 8.63E-08 |
| Dystonin | Q91ZU6 | 0.54 | 1 | 5.89E-07 |
| Kinesin-like protein KIF21A | Q7Z4S6 | 0.92 | 1 | 1.07E-07 |
| Annexin A11 | P97384 | 13.4 | 3 | 0.000474185 |
| Superoxide dismutase [Cu-Zn] | O22373 | 7.53 | 1 | 0.000591094 |
| Uncharacterized protein | V4AZ85 | 3.2 | 1 | 2.27E-05 |
| Prolow-density lipoprotein receptor-related protein 1 | Q91ZX7 | 0.35 | 1 | 2.32E-09 |
| Early endosome antigen 1 | Q15075 | 1.11 | 1 | 0.003817603 |
| Zinc finger protein 454 | Q8N9F8 | 1.4 | 1 | 5.31E-06 |
| Allograft inflammatory factor 1-like | Q9BQI0 | 18.5 | 2 | 2.33E-06 |
| Epidermal growth factor receptor | P0CY46 | 1.08 | 1 | 8.62E-09 |
| Fumarylacetoacetate hydrolase domain-containing protein 2 | Q6GLT8 | 2.92 | 2 | 2.23E-08 |
| Tryptophan--tRNA ligase, cytoplasmic | Q5R4J1 | 6.48 | 3 | 1.91E-05 |
| Myosin regulatory light chain sqh | P40423 | 11.7 | 2 | 3.68E-08 |
| Putative aminopeptidase W07G4.4 | Q27245 | 25.9 | 15 | 0.000103036 |
| **protein binding** | | | | |
| Mitochondrial import receptor subunit TOM70 | Q75Q39 | 2.01 | 1 | 0.000884971 |
| Putative ankyrin repeat protein RF_0381 | Q4UMH6 | 0.63 | 1 | 0.015986458 |
| Serine/threonine-protein phosphatase 6 regulatory ankyrin repeat subunit C | Q5ZLC8 | 3.2 | 1 | 0.005402656 |
| Large proline-rich protein BAG6 | P46379 | 0.54 | 1 | 6.95E-09 |
| Glutathione S-transferase Mu 2 | P15626 | 34.9 | 8 | 8.44E-06 |
| Lymphocyte cytosolic protein 2 | Q13094 | 1.35 | 1 | 0.000484509 |
| Uncharacterized protein (Fragment) | A0A0B7ALS3 | 2.73 | 1 | 1.44E-05 |
| Transgelin | K1PFT9 | 7.41 | 2 | 1.98E-06 |
| Semaphorin-5A | D3ZTD8 | 1.24 | 1 | 1.36E-08 |
| Nesprin-1 | Q8NF91 | 0.14 | 1 | 3.87E-05 |
| Hemicentin-1-like | A0A0G2AJP4 | 1.75 | 1 | 1.99E-05 |
| **hydrolase activity** | | | | |
| Cathepsin C | A0A023PJH7 | 2.22 | 1 | 4.13E-06 |
| Palmitoyl-protein thioesterase 1 | Q8HXW6 | 4.67 | 1 | 4.57E-05 |
| Acetylcholinesterase | Q86GC8 | 1.77 | 1 | 4.28E-06 |
| Glycerophosphodiester phosphodiesterase domain-containing protein 1 | Q8N9F7 | 2.5 | 1 | 5.66E-05 |
| Cathepsin B | A1E295 | 3.27 | 1 | 3.84E-08 |

**Table S3.** *cont.*

| **Protein Description** | **Accession Number** | **Coverage (%)** | **Peptide** | ***p*-value** |
| --- | --- | --- | --- | --- |
| **hydrolase activity** | | | | |
| N-acetylglucosamine-6-sulfatase | Q8BFR4 | 3.16 | 1 | 0.000222518 |
| **transport** | | | | |
| Aquaporin-4 | P55088 | 6.94 | 1 | 4.91E-06 |
| Glutamate receptor 4 | Q9Z2W8 | 2.08 | 1 | 0.000158175 |
| **transferase activity** | | | | |
| UDP-glucose:glycoprotein glucosyltransferase 1 | Q9JLA3 | 0.89 | 1 | 1.50E-06 |
| Carbohydrate sulfotransferase 11 | Q9JME2 | 1.17 | 1 | 1.47E-05 |
| Thymidine phosphorylase | Q5FVR2 | 26.3 | 10 | 8.78E-08 |
| Phosphatidylinositol 5-phosphate 4-kinase type-2 beta | P78356 | 1.74 | 1 | 0.006620131 |
| **primary metabolic process** | | | | |
| Ganglioside GM2 activator | Q8HXX6 | 8.04 | 2 | 1.78E-06 |
| Alpha-N-acetylgalactosaminidase | Q90744 | 6.68 | 2 | 1.28E-05 |
| DNA topoisomerase 3-beta-1 | O95985 | 1.62 | 1 | 0.000240282 |
| **intracellular** | | | | |
| Histone H4 | P0CG89 | 39.8 | 4 | 1.11E-07 |
| Rho GTPase-activating protein 44 | Q5SSM3 | 2.57 | 1 | 0.010312344 |
| Dynein beta chain, flagellar outer arm | Q39565 | 0.22 | 1 | 0.002348174 |
| **response to stress** | | | | |
| Putative universal stress protein SSP1056 | Q49YE0 | 5.96 | 1 | 4.21E-07 |
| **ligase activity** | | | | |
| E3 ubiquitin-protein ligase NEDD4 | P46935 | 1.71 | 1 | 0.000475396 |
| **cofactor binding** | | | | |
| Dihydropteridine reductase | P11348 | 3.8 | 1 | 1.05E-06 |
| **cargo receptor activity** | | | | |
| Neurotrypsin | Q5G266 | 1.83 | 1 | 8.42E-06 |
| **integral component of membrane** | | | | |
| CD9 antigen | P40241 | 5.76 | 1 | 9.28E-07 |
| **extracellular space** | | | | |
| Serpin B8 | P50452 | 20.2 | 5 | 2.47E-06 |
| **single-organism metabolic process** | | | | |
| Aldehyde dehydrogenase, mitochondrial | P20000 | 6.88 | 1 | 0.011485455 |

**Table S3.** *cont.*

| **Protein name** | **Accession Number** | **Coverage (%)** | **Peptide** | ***p*-value** |
| --- | --- | --- | --- | --- |
| **Unknown function protein** | | | | |
| Alpha-aminoadipic semialdehyde synthase, mitochondrial | A8E657 | 2.85 | 1 | 6.00E-07 |
| Uncharacterized protein | V4A573 | 3.19 | 1 | 1.71E-06 |
| Kitasatospora griseola strain MF730-N6 RKJC_4, whole genome shotgun sequence | A0A0D0PVQ3 | 11.4 | 1 | 0.00014492 |
| Collagen alpha-4(VI) chain | A2AX52 | 12.1 | 15 | 1.06E-05 |
| Beta actin | G8HY07 | 60.5 | 1 | 1.31E-12 |
| Uncharacterized protein | V3ZES3 | 0.31 | 1 | 4.58E-05 |
| Type I inositol 3,4-bisphosphate 4-phosphatase | Q62784 | 0.95 | 1 | 0.001976714 |
| 3-hydroxyacyl-CoA dehydrogenase type-2 | O18404 | 4.38 | 1 | 0.000197383 |
| Collagen alpha-3(IX) chain | P32017 | 3.16 | 1 | 0.000721883 |
| Uncharacterized oxidoreductase C663.09c | Q7Z9I2 | 3.14 | 1 | 0.00032111 |
| - | - | 9.86 | 1 | 1.41E-06 |
| cGMP-inhibited 3',5'-cyclic phosphodiesterase A | Q14432 | 2.97 | 2 | 1.23E-09 |
| Acetyl-CoA acetyltransferase, cytosolic | Q8CAY6 | 10.2 | 1 | 6.09E-05 |
| Opioid growth factor receptor | Q99PG2 | 1.24 | 1 | 9.21E-12 |
| Protein furry homolog | Q5TBA9 | 0.69 | 1 | 1.22E-05 |
| Retinol dehydrogenase 13 | Q8NBN7 | 3.24 | 4 | 1.94E-05 |
| Profilin | F4XXT7 | 19 | 1 | -0.688881449 |
| - | - | 16.1 | 1 | 7.91E-06 |
| Uncharacterized protein | V4C7P8 | 1.32 | 1 | 0.000612706 |
| Uncharacterized protein | G6CQ42 | 4.58 | 1 | 5.55E-07 |
| Cilia- and flagella-associated protein 54 | Q96N23 | 0.27 | 1 | 0.00098378 |
| Trans-aconitate 2-methyltransferase | B0UPF4 | 11.3 | 3 | 8.07E-08 |
| Putative uncharacterized protein (Fragment) | B3TK33 | 2.8 | 1 | 3.39E-05 |
| Predicted protein (Fragment) | A7SIW6 | 2.58 | 1 | 1.58E-06 |
| Uncharacterized protein | V4A8S4 | 1.41 | 1 | 1.30E-06 |
| Fibrinogen-like protein A | P19477 | 7.03 | 2 | 8.07E-06 |

| **Table S4**. Primers used for qRT-PCR. | | |
| --- | --- | --- |
| Gene name | Forward | Reverse |
| *MYH* | GACCCCAACGACCCTGATAT | TCTTCTCCCTTGGTGCTCTG |
| *myophilin* | CCAGGTCACGCTCTGTATC | GGCTCACGATGTTCTTGGC |
| *SLC2A3* | GTGGTCTTCAACTGTGGCTG | CTCTGGAGACTTGGCGATGA |
| *CTSC* | CTGGTGAAGAACGGACCTGT | GCTCCATACCCTACGACCAG |
| *profilin* | ACGGCAACTCCTGGACAAG | CACTCTGGGCTTTGGTCGT |
| *EGFR* | AGAACATTGACCCCTCCAGG | ACCATCACACCACCAATTGC |
| *18S rRNA* | TTCCCAGTAAGCGTCAGTCATC | CGAGGGTCTCACTAAACCATTC |

**
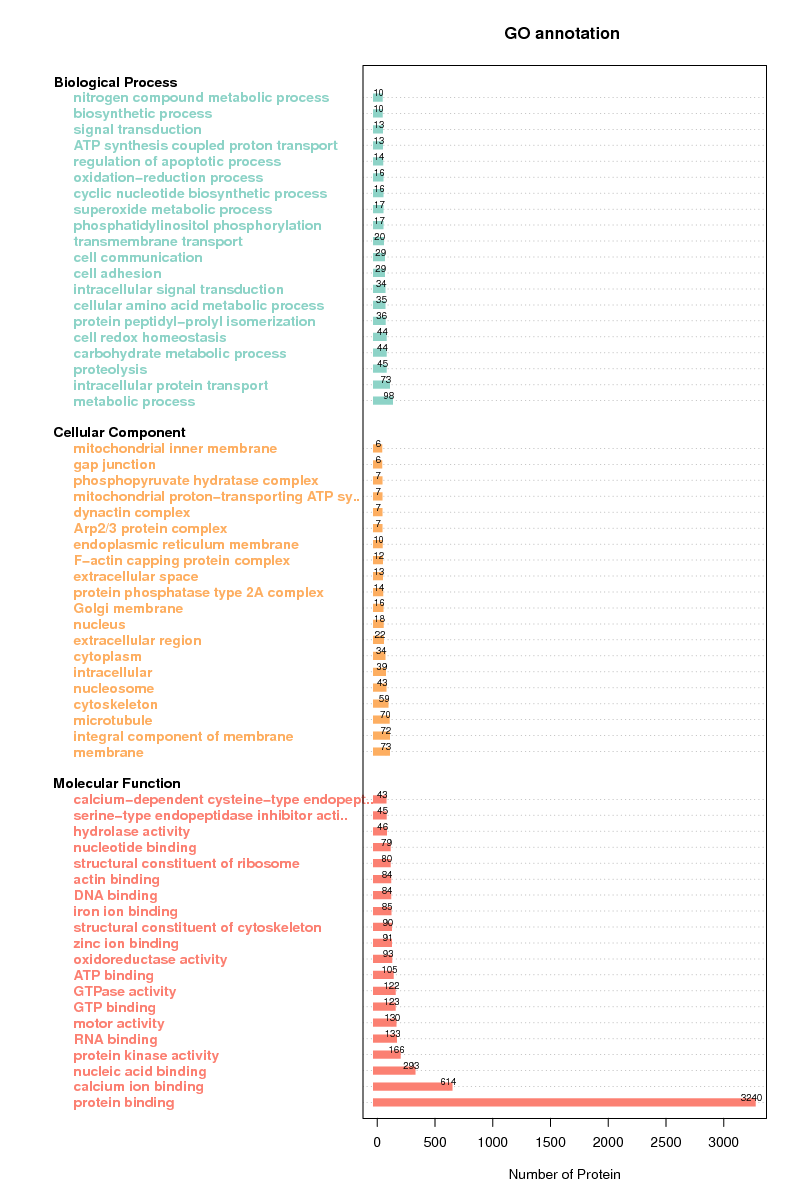
**

**Figure. S1.** Gene ontology (GO) analysis of proteins in *Haliotis discus hannai* on the basis of biological process, cellular component and molecular function.
